# Intrinsic capacity differs from functional ability in predicting 10-year mortality and biological features in healthy aging: results from the I-Lan longitudinal aging study

**DOI:** 10.18632/aging.204508

**Published:** 2023-02-06

**Authors:** Wei-Ju Lee, Li-Ning Peng, Ming-Hsien Lin, Ching-Hui Loh, Fei-Yuan Hsiao, Liang-Kung Chen

**Affiliations:** 1 Center for Healthy Longevity and Aging Sciences, National Yang Ming Chiao Tung University, Taipei, Taiwan; 2 Department of Geriatric Medicine, National Yang Ming Chiao Tung University, School of Medicine, Taipei, Taiwan; 3 Department of Family Medicine, Taipei Veterans General Hospital Yuanshan Branch, Yi-Lan County, Taiwan; 4 Center for Geriatrics and Gerontology, Taipei Veterans General Hospital, Taipei, Taiwan; 5 Center of Health and Aging, Hualien Tzu Chi Hospital Buddhist Tzu Chi Medical Foundation, Hualien County, Taiwan; 6 Graduate Institute of Clinical Pharmacy, College of Medicine, National Taiwan University, Taipei, Taiwan; 7 School of Pharmacy, College of Medicine, National Taiwan University, Taipei, Taiwan; 8 Department of Pharmacy, National Taiwan University Hospital, Taipei, Taiwan; 9 Taipei Municipal Gan-Dau Hospital, Taipei, Taiwan

**Keywords:** intrinsic capacity, functional ability, healthy aging, mortality, biomarkers

## Abstract

This study aimed to explore the biological features and mortality risk of intrinsic capacity (IC) and functional ability (FA). Based on data from 1839 participants from the I-Lan Longitudinal Aging Study, multivariable Cox proportional hazard models were used to evaluate the predictive ability of IC (range 0–100) and FA (range 0–100) on 10-year mortality. Of 2038 repeated measurements for IC within a 7-year observational period, multivariable logistic regression was used to compare biological features of participants with maintained, improved and rapidly deteriorated IC. A 1-point increased IC value was associated with a 5% (HR 0.95, 95% CI 0.93–0.97, *p* < 0.001) decrease in mortality risk. Low IC (HR 1.94, 95% CI 1.39–2.70, *p* < 0.001) was associated with higher mortality risk. Hyperglycemia (OR 1.40, 95% CI 1.09–1.81, *p* = 0.010), low serum levels of DHEA-S (OR 3.33, 95% CI 1.32–8.41, *p* = 0.011), and high serum levels of C-reactive protein (OR 1.45, 95% CI 1.05–2.00, *p* = 0.023) were associated with low IC at baseline. Low serum levels of DHEA-S (middle tertile OR 1.84, 95% CI 1.15–2.95, *p* = 0.012; lowest tertile OR 2.25, 95% CI 1.34–3.77, *p* = 0.002) and vitamin D deficiency (OR 1.82, 95% CI 1.02–3.27, *p* = 0.044) were associated with rapid deterioration of IC. IC and FA predicted 10-year mortality, whereas chronic inflammation, hyperglycemia, and low DHEA-S were associated with low IC status. Low DHEA-S and vitamin D deficiency may be considered as potential biomarkers of rapid IC declines, which implies underlying biological mechanisms of healthy aging.

## INTRODUCTION

The World Health Organization (WHO) published the World Report on Aging and Health in 2015 and the Integrated Care for Older People (ICOPE) in 2017, which has transformed health services from the traditional disease-focused approach into a function-centered one in the scheme of healthy aging. Healthy aging is defined as the process of developing and maintaining functional ability to ensure wellbeing in later life; IC and FA are proposed to describe and estimate the state of healthy aging [1, 2]. IC, defined as a composite measure for all physical and mental capacities of an individual, is conceptualized as a dynamic construct to serve as the potential functional reserve in the aging process [3]. Interacting with environmental factors, such as facilitators or barriers, IC may be a proxy to estimate the FA of an individual and the status of healthy aging [1]. Notably, as conceptualized, IC declines occur earlier before clinical manifestations of FA declines, so it is critical to capture IC declines in the life course for healthy longevity [4].

In the WHO Integrated Care for Older People, IC consists of five elements, i.e., locomotion, sensory, vitality, psychological, and cognition, which represent the physiological competence of individuals to support their FA [1]. Although there are strong theoretical and consensual bases to support this multidomain conceptualization of IC [5–8], most empirical studies have focused on the associations between IC and disability [4, 9], falls [10], quality of life [11], and mortality [12–14]. Unique diet, lifestyle, culture and policy may interact with IC, and contribute to FA, particularly in Asia [8]. In this context, functioning was used as a target to build an IC model instead of exploring the role of FA on health outcomes. Nevertheless, questions remained as there might be a gap between IC and FA against mortality due to their different conceptualization regarding a person’s competency and ability. To the best of our knowledge, no study has examined the impacts of these two distinct constructs on mortality in parallel to distinguish their potential impacts on clinical outcomes. A systemic review exploring possible biomarkers related to aging showed that lipids, glucose, inflammatory biomarkers, dehydroepiandrosterone sulfate (DHEA-S), growth hormone and insulin-like growth Factor 1 (IGF-1) were candidate biomarkers [15, 16]. Other studies indicated that elevated systemic inflammatory biomarkers, such as high-sensitivity C-reactive protein (hsCRP) and homocysteine, were associated with slow gait speed, weak muscle strength, and low IC [17, 18]. However, conflicting results have been reported regarding the associations between specific biomarkers and IC or FA. Although the underlying biological mechanisms of healthy aging remain unclear, identifying the biological features of healthy aging helps to capture the heterogenicity of aging over time [19]. Moreover, assessing the biological features of IC enables researchers and clinicians to understand potential pathophysiological mechanism, and to design personalized intervention programs to promote healthy aging.

To address the abovementioned knowledge gaps, the study aimed 1) to examine the association between declines in IC or FA and 10-year mortality risk and 2) to further explore the biological features of IC based on its longitudinal changes.

## RESULTS

### Demographic characteristics of participants stratified by IC and FA status

The demographic characteristics of the participants (mean age 63.9 ± 9.3 years, 47.5% men) are shown in Supplementary Table 1. Compared to the high IC group (*n* = 1190), those with low IC (*n* = 649) were older (70.0 ± 8.8 vs. 60.6 ± 7.7 years old), mainly women (63.0% vs. 46.8%), had fewer years of education (2.5 ± 3.4 vs. 8.2 ± 4.5 years), were less likely to consume alcohol (23.0% vs. 38.5%), and had a higher disease burden (CCI 1.6 ± 1.4 vs. 0.7 ± 1.1). Compared to the high FA group (*n* = 1769), those with low FA (*n* = 70) were older (76.0 ± 9.9 vs. 63.5 ± 8.9 years old), had fewer years of education (2.9 ± 4.1 vs. 6.4 ± 4.9 years), were less likely to consume alcohol (15.7% vs. 33.7%) and had a higher disease burden (CCI 2.3 ± 1.6 vs. 1.0 ± 1.2). Comparisons of subcategories of IC were shown in Supplementary Table 2. The distributions of IC and FA were left-skewed (Supplementary Figure 1). Overall, IC declined progressively from 86.0 ± 5.1 to 80.5 ± 5.1 points, and FA declined from 99.9 ± 1.3 to 99.3 ± 4.1 points in a mean follow-up period of 6.5 ± 0.8 years.

### Survival analysis for IC and FA status

**Figure 1:**
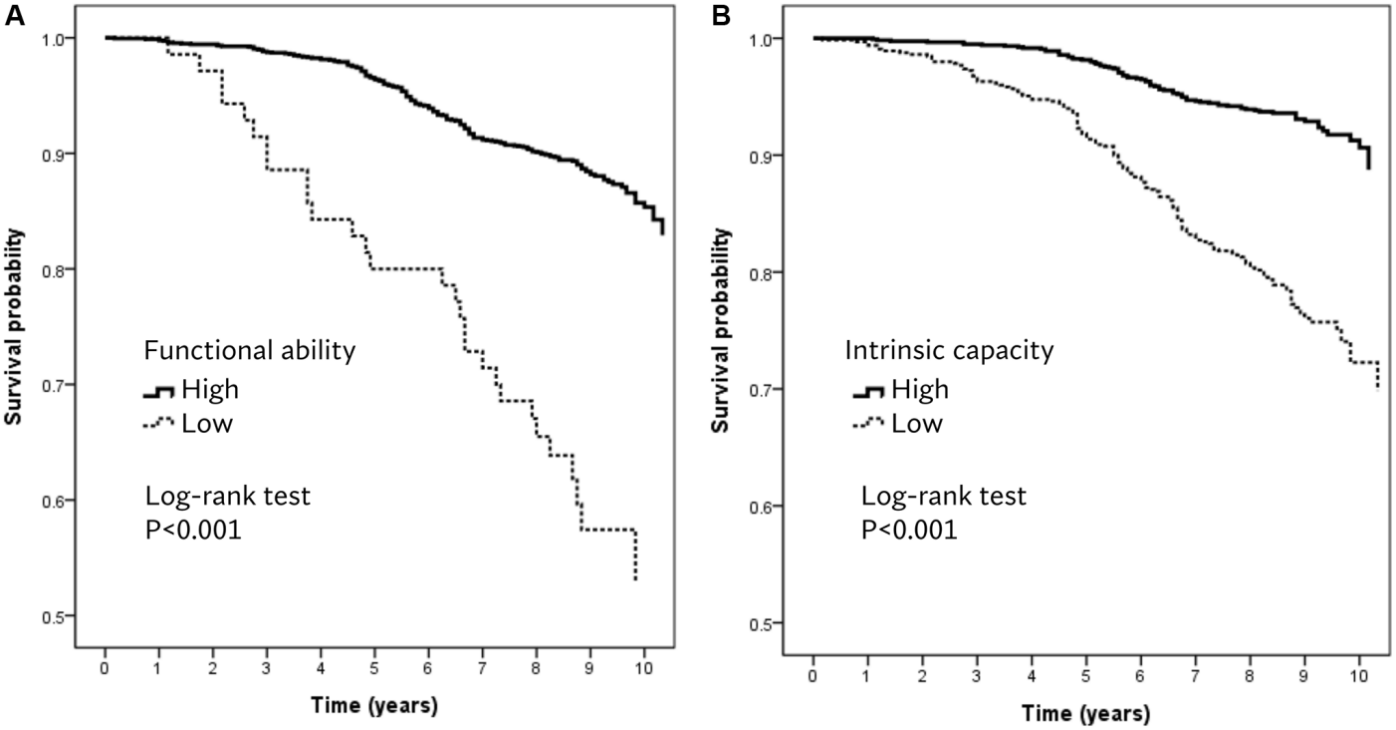
Kaplan–Meier survival plots for (**A**) functional ability and (**B**) intrinsic capacity.

There were 238 deaths in a mean follow-up period of 8.5 ± 1.5 years. Kaplan–Meier analysis showed that low FA (Figure 1A, log-rank test, *p* < 0.001) and low IC (Figure 1B, log-rank test, *p* < 0.001) were significantly associated with mortality. A one-point (percent) increase in IC score decreased the odds of mortality by 5% (HR 0.95, 95% CI 0.93–0.97, *p* < 0.001), and those with low IC had a greater risk of mortality (HR 1.94, 95% CI 1.39–2.70, *p* < 0.001). The associations between FA and mortality were attenuated after adjusting for relevant confounders (Table 1).

**Table 1:** Relationship between Intrinsic capacity, functional ability and mortality.

	**Model I^a^**	***p***	**Model II^b^**	***p***
	**HR (95%CI)**		**HR (95%CI)**	
Intrinsic capacity^c^	0.94 (0.92–0.96)	<0.001	0.95 (0.93–0.97)	<0.001
Functional ability^c^	0.97 (0.94–1.00)	0.025	0.97 (0.94–1.00)	0.089
Low Intrinsic capacity^d^	2.19 (1.58–3.04)	<0.001	1.94 (1.39–2.70)	<0.001
Low Functional ability^d^	1.53 (1.00–2.33)	0.049	1.29 (0.84–1.97)	0.248
Components of intrinsic capacity^c^				
Locomotion	0.97 (0.96–0.98)	<0.001	0.97 (0.96–0.98)	<0.001
Cognition	0.99 (0.98–1.00)	0.010	0.99 (0.98–1.00)	0.018
Psychology	0.99 (0.97–1.00)	0.022	0.99 (0.98–1.00)	0.139
Vitality	0.97 (0.95–0.98)	<0.001	0.98 (0.96–0.99)	0.006
Sensory	0.98 (0.95–1.00)	0.047	0.98 (0.96–1.01)	0.182

^a^Cox proportional hazards model adjusted for age, gender, and education years. ^b^Cox proportional hazards model adjusted for age, gender, education years, smoke, drink and charlson comorbidity index. ^c^numerical variables. ^d^categorical variables.

### Biomarkers associated with low IC

**Figure 2:**
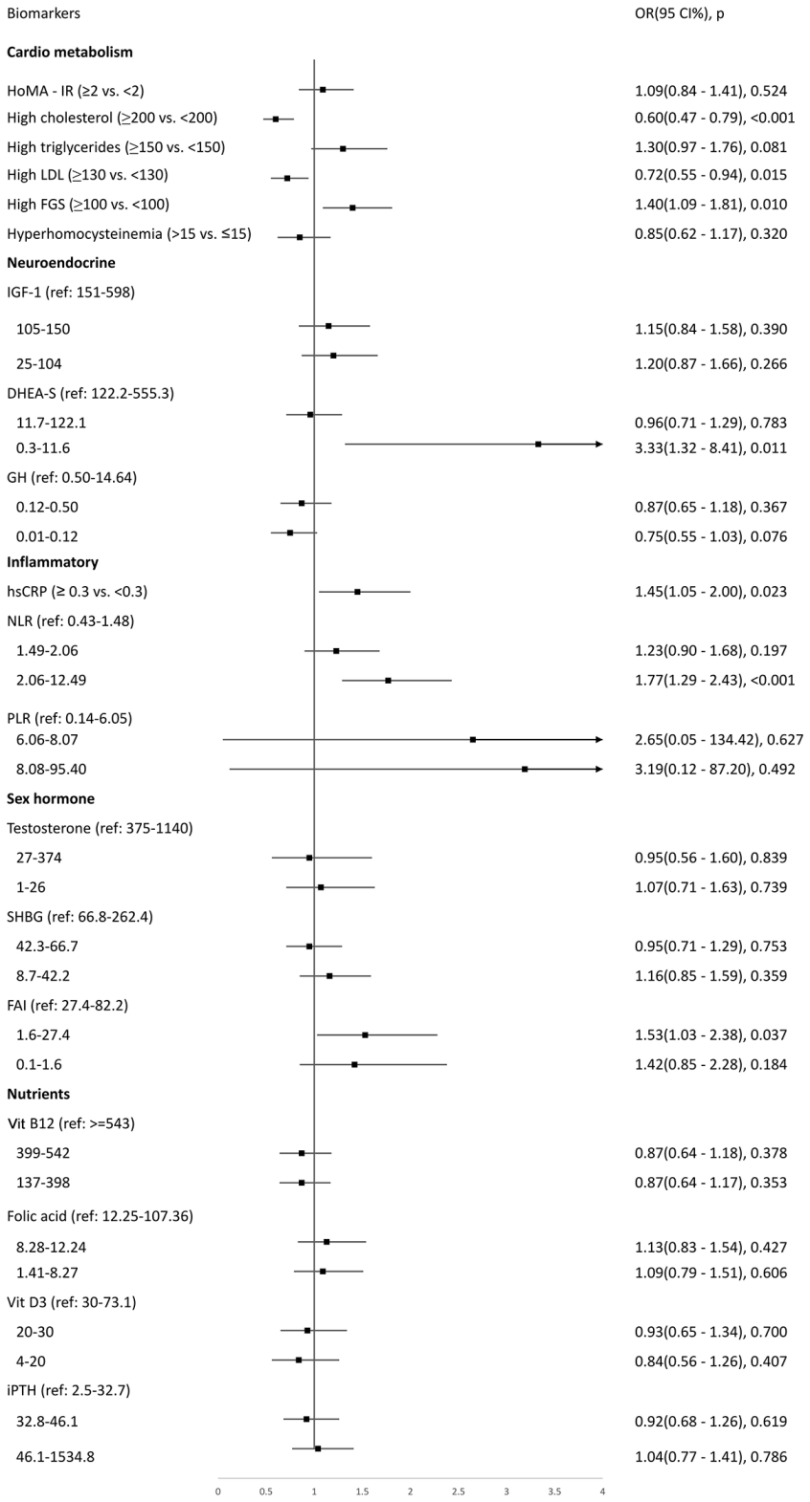
Logistic regression to explore biomarkers associated with low intrinsic capacity at baseline.

The results of adjusted logistic regression analysis for the associations between biomarkers and low IC are shown in Figure 2. Participants with higher levels of fasting glucose had higher odds of having low IC (OR 1.40, 95% CI 1.09–1.81, *p* = 0.010), but those with higher levels of cholesterol (OR 0.60, 95% CI 0.47–0.79, *p* < 0.001) and LDL-C (OR 0.72, 95% CI 0.55–0.94, *p* = 0.015) were less likely to have low IC. Those in the lowest tertile of DHEA-S had higher odds for low IC (OR 3.33, 95% CI 1.32–8.41, *p* = 0.011). Of the inflammatory biomarkers, higher levels of hsCRP (OR 1.45, 95% CI 1.05–2.00, *p* = 0.023) and neutrophil-to-lymphocyte ratio (NLR, highest tertile OR 1.77, 95% CI 1.29–2.43, *p* < 0.001) were significantly associated with low IC (Figure 2).

### Biomarkers associated with longitudinal preservation or deterioration of IC

**Figure 3:**
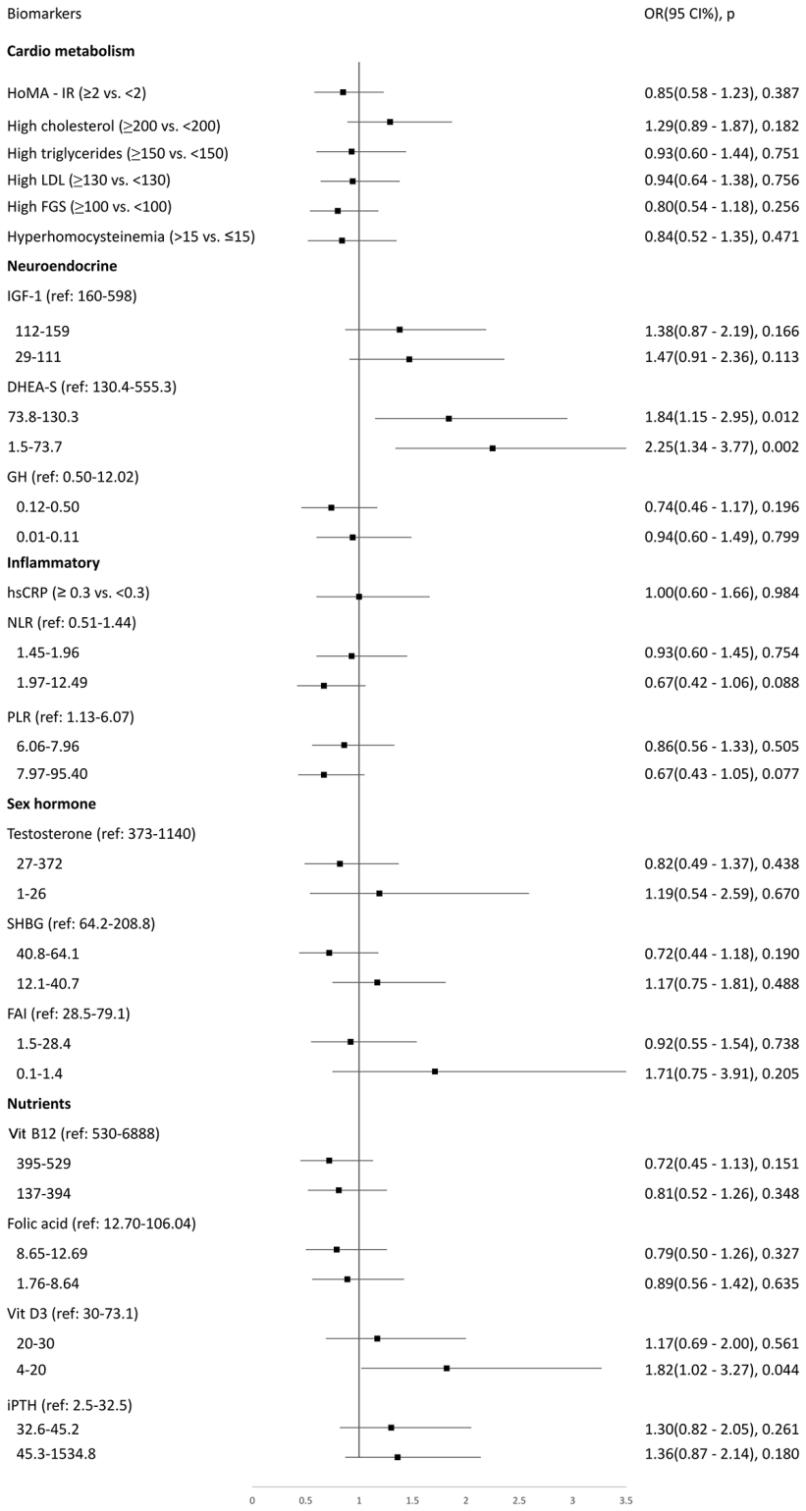
Logistic regression to explore biomarkers associated with rapidly deteriorated intrinsic capacity at a 7-year follow-up period.

Adjusted logistic regressions showed that predictors for rapid deterioration of IC were lower levels of dehydroepiandrosterone sulfate (DHEA-S, middle tertile OR 1.84, 95% CI 1.15–2.95, *p* = 0.012; lowest tertile OR 2.25, 95% CI 1.34–3.77, *p* = 0.002) and vitamin D deficiency (OR 1.82, 95% CI 1.02–3.27, *p* = 0.044) (Figure 3). Inverse probability weighting regressions were used to reduce potential selection bias from excluded participants, and findings were similar (OR 1.41, 95% CI 1.09–1.83, *p* = 0.008 for lowest tertile of DHEA-S; OR 1.06, 95% CI 1.01–1.12, *p* = 0.030 for vitamin D deficiency). Supplementary Figure 2 shows adjusted logistic regression to explore potential biomarkers for maintained or improved IC. All biomarkers showed insignificant associations.

## DISCUSSION

The WHO conceptualized the scheme of healthy aging and addressed the importance of IC and FA in the life course that shifted health care services from disease-centric models to function-centric ones, as well as the focus on positive attributes of health. This study used a longitudinal cohort to compare the 10-year mortality risk between IC and FA. Unlike IC, the mortality risk of FA attenuated after adjustment for smoking, drinking, and disease burden, which supports the idea that environmental factors modify health outcomes in older age. However, the constant mortality risk of IC clearly reflects its adverse impacts on health outcomes in the aging process. In addition, this study found that hyperglycemia, proinflammatory status of hsCRP and NLR, and low serum levels of DHEA-S were associated with low IC. In particular, lower serum levels of DHEA-S and vitamin D deficiency were associated with rapid deterioration of IC. These findings suggest the potential roles of inflammation and endocrine and musculoskeletal systems in healthy aging through their influences on IC.

Based on the conceptual framework of healthy aging, the impacts of environmental or modifiable factors, including interventions on diseases and healthy behaviors, were emphasized to constitute FA instead of focusing on age-related declines in IC. The results of the current study completely support the conceptual framework of healthy aging. In contrast to frailty characterized by increased vulnerability to stressors from disrupted homeostasis of multiple physical systems, IC aimed to capture the nature of physiological reserves and residual capacities in aging, which is particularly suitable for longitudinal assessments [3]. Although frailty and IC are two interrelated but distinct constructs, both of them focus on function-centric health services with comprehensive assessment and are provided in a multidisciplinary fashion. Our previous study indicated that multidomain interventions combined with integrated primary care significantly improve physical function, cognitive performance, and quality of life among older adults with multimorbidity, which supported the core concepts of healthy aging [20, 21]. Nevertheless, the importance of FA should be addressed as well because of the significant modifying effects of environmental factors in older age.

By examining associations between IC and biomarkers, this study disclosed the multidimensional nature of biological mechanisms in the process of healthy aging. In this study, low IC status was associated with glucose metabolism, chronic inflammation, and neuroendocrine diseases. Previous studies have suggested associations between hsCRP, NLR, poor locomotion and vitality [17, 22]. This study further extended these associations from two specific domains (locomotion and vitality) to the whole composite IC, which strengthens the roles of inflammation in the aging process. On the other hand, cholesterol and LDL-C are well-known cardiovascular risk factors, but the associations are attenuated in late life [23]. A systemic review concluded that lower serum cholesterol levels were associated with greater mortality risk in older adults [24], which is in line with our study that lower cholesterol and low-density lipoprotein cholesterol (LDL-C) increased the risk of poor IC. These data justified the debates on statin treatment in older adults that routine statin use was not encouraged for those aged 75 years old and older. In addition, deprescription of statin therapy in those who have developed frailty is usually recommended [25]. Hyperglycemia is also a factor contributing to incident frailty and disability [26], which explained its association with low IC in our study.

DHEA-S is being widely used in the general public to prevent age-related disease. However, oral supplementation with DHEA-S failed to demonstrate a positive impact on the improvement of cognitive performance in healthy older people [27], whereas it preserved bone health [28]. Vitamin D is crucial to musculoskeletal health [29], but a systemic review of 81 randomized controlled trials reported disappointing results on falls, fracture preventions, or meaningful effects on bone mineral density [30]. Previous studies have suggested potential associations between DHEA-S and inflammatory [31] as well as the benefits for DHEA-S supplement administration on libido improvement and bone turnover biomarkers improvement [32]. In this study, low DHEA-S and vitamin D deficiency were both associated with rapid deterioration of IC but were not associated with maintained or improved IC in a follow-up period of up to 7 years. These findings highlight the importance of musculoskeletal health in preventing IC decline but also reflect the fact that neither monodimensional factor could counter the effects of age-related IC decline stemming from deficits in multiple physiological systems. Further interventional studies to explore the effects of oral supplementation with DHEA-S and vitamin D on IC are needed to further guide the optimal use of these supplements.

Despite all efforts made in this study, there are some limitations to note. First, environmental factors such as access to health facilities or means of transportation, etc., could not be adjusted for FA due to the limitation of data availability. Nevertheless, FA measured by the Functional Autonomy Measurement System (SMAF) considered available resources and environmental factors to compensate for functional impairment. Second, information regarding the cause of death could not be available in this study, which precludes cause-specific analysis. Further biological marker analysis could provide information on IC decline and potential mechanisms. Third, those excluded from the second part of this study might introduce selection bias. However, we have conducted an inverse probability weighting analysis to adjust for such potential selection bias and yields similar results. Fourth, as the participants from ILAS were community-dwelling adults, they are relative healthy. That is why the cutoff value of functional ability was quite high. Last, subdomains of vision and hearing were obtained from self-report questionnaires, which may probably underestimate the prevalence of vision and hearing impairment.

In conclusion, IC and FA predicted 10-year mortality, whereas the association for FA diminished after adjusting for smoking, drinking, and disease burden. Chronic inflammatory markers of hsCRP and NLR, hyperglycemia, and low DHEA-S were associated with low IC status. DHEA-S and vitamin D deficiency aggravated IC deterioration, which implies the importance of musculoskeletal health in healthy aging.

## MATERIALS AND METHODS

### Participants and study design

**Figure 4:**
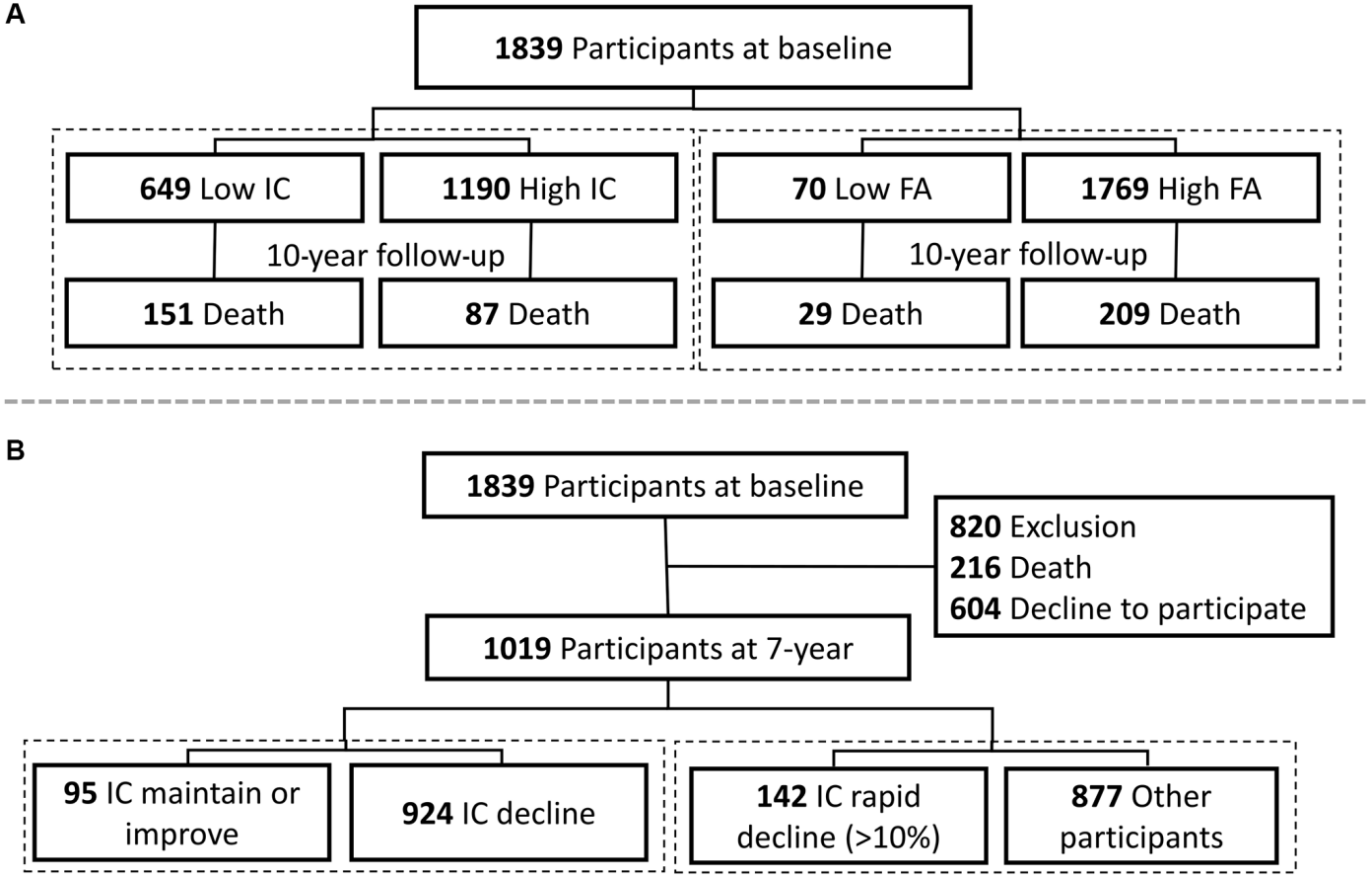
Study flowcharts of (**A**) survival analysis and (**B**) longitudinal study for biological features of intrinsic capacity. Abbreviations: IC: denotes intrinsic capacity; FA: denotes functional ability.

Data from the first and third waves of the I-Lan Longitudinal Aging Study (ILAS) were collected for the baseline cross-sectional (wave 1) and longitudinal cohort analyses (wave 1 and 3). ILAS was a prospective cohort study focused on interactions between sarcopenia, frailty, and cognitive functioning throughout the aging process. The details of the study design, participant recruitment, and data collection of ILAS have been reported previously [33]. Briefly, ILAS recruited community-dwelling adults aged ≥50 years without severe disability, dementia or communication difficulties, limited life expectancy due to major illness or being institutionalized. The first part of this study was a survival analysis of a 10-year follow-up, and the second part was a 7-year longitudinal study to capture biological features based on the status of IC changes (maintained, improved and rapidly deteriorated). In the first part of the study, data from 1,839 participants from the ILAS wave 1 survey in 2011 were used to evaluate the 10-year mortality risk of IC and FA (Figure 4A). In the second part of the study, 1019 participants who recruited in ILAS wave 1 and completed the ILAS wave 3 survey since 2018 (216 participants died before wave 3 survey and 604 participants declined to participate in wave 3 survey) were enrolled (Figure 4B). Compared to those enrolled in the second part of this study, those excluded were older (67.5 ± 10.0 vs. 61.1 ± 7.5 years, *p* < 0.001), less educated (4.5 ± 4.5 vs. 7.6 ± 4.8 years, *p* < 0.001), higher disease burden of Charlson comorbidity index (1.4 ± 1.4 vs. 0.7 ± 1.1, *p* < 0.001), but similar sex proportions.

This study was designed and conducted in accordance with the principles of the 1964 Declaration of Helsinki and later amendments. The institutional review board of National Yang Ming University (YM103008) and Taipei Veterans General Hospital (2018-05-003B) approved the study protocol, and written informed consent was obtained from each individual before inclusion. The observational design and reporting format followed the STROBE guidelines [34].

### Intrinsic capacity (IC)

The five IC elements, i.e., cognition, locomotion, vitality, psychology, and sensory, were selected based on the principle proposed by WHO ICOPE [5].

Cognition was evaluated by the Chinese version of the Mini-Mental State Examination (MMSE), with scores ranging from 0 to 30, and a higher score on the MMSE indicated better cognition [35]. Locomotion was assessed by a timed 6-meter gait speed at the usual pace in meters/second based on a consensus recommended by the Asian Working Group for Sarcopenia (AWGS) [36]. Vitality was assessed by the Mini Nutritional Assessment (MNA), with scores ranging from 0 to 30, and a higher score indicated better nutrition [37]. Psychology was measured with the Center for Epidemiologic Studies—Depression scale (CESD) with scores ranging from 0 to 60, and a higher score denoted a greater level of depressive symptoms [38]. Psychological scores were obtained by multiplying the original CESD scores by −1 in this study. The reason why we would like to adopt this approach is mainly because IC was constructed based on positive capacity for physical and mental reserves. However, we used CESD to measure mental reserve, but a higher CESD score indicates the higher level of depression. We thus rescale our CESD by multiplying the original CESD by −1. Sensory impairment was assessed through a self-reported score comprising visual and hearing impairments based on a previous study [12]. A sensory score was built from two questions of vision and hearing: score from 0 (independent), −1 (need supervision), −2 (need help), and −3 (dependent), yielding a total score ranging from −6 to 0. We further used the percent of maximum possible method - calculated by [100 × (observed − minimum)/(maximum − minimum)]- to rescale all individual variables with a ranging from 0 (minimum possible) to 100 (maximum possible) [39] to make the composite values of IC comparable longitudinally [40]. Psychological scores were obtained by 100 minus the rescaled CESD scores. For each study participant, we calculated their IC scores as the mean of the sum of subscores obtained in each of five elements.

### Functional ability (FA)

Functional ability (FA) was measured by SMAF through the percent of maximum possible method to rescale the original score with the range from 0 to 100. SMAF is a 29-item four-level measurement scale based on WHO’s classification of impairments, disabilities, and handicap with consideration of available resources and environmental factors to compensate for functional impairment [41]. The SMAF assessed functional ability in 5 areas: activities of daily living (ADL) [7 items], mobility [6 items], communication [3 items], mental functions [5 items] and instrumental activities of daily living (IADL) [8 items]. For each item, the disability was scored on a 4-level scale: 0 (independent), −0.5 (with difficulty), −1 (needs supervision), −2 (needs help), −3 (dependent). Resources available to compensate for the disability were also evaluated, and a handicap score was deducted. The stability of the resources was also assessed. A disability score (on −87) can be calculated, together with subscores for each dimension. Given the involvement of environmental factors, the SMAF evaluated a person’s FA rather than their IC alone [42].

### Acquisition of mortality

All participants in ILAS received a phone call by research nurses every 3 months to check their health conditions and survival status. These survival data were calculated from the index interview day until the last phone contact before 31 March 2022.

### Other variables

Potential confounding variables were identified from previous literature, which included age, sex, education years, smoking and alcohol consumption in the past six months (yes versus no). The burden of disease was assessed by Charlson’s comorbidity index (CCI) [43]. Venous blood samples were collected from all ILAS participants after a 10-hour overnight fast. Biomarkers related to cardiometabolic health, hormones and biochemistry were tested for all participants, including fasting glucose, total cholesterol, triglycerides, LDL, insulin level, and homeostasis model assessment-insulin resistance (HOMA-IR)-insulin resistance. Inflammatory biomarkers (NLR, platelet-to-lymphocyte ratio, homocysteine, and hsCRP), age-related hormones (growth hormone, IGF-1, DHEA-S, testosterone, sex hormone binding globulin (SHBG), intact parathyroid hormone, and micronutrients (vitamin B12, folic acid and vitamin D, measured by 25-OH vitamin D) were also tested for all participants. Details of the machine, limit of detection, and intra- and inter-assay coefficients of variation for serum biomarkers are shown in the appendix (Supplementary Table 3). The free androgen index (FAI) was calculated from testosterone and SHBG [44].

### Statistical analysis

Numerical variables were expressed as the mean ± standard deviation, and categorical variables were expressed as numbers with percentages. Comparisons of baseline characteristics between different IC or FA groups were performed by Student’s *t* test for continuous variables and chi-square tests or Fisher’s exact test for categorical variables. In the first part of this study, IC (≥82.7 vs. <82.7) and FA (≥98.9 vs. <98.9) at baseline were assigned to high IC/FA or low IC/FA groups according to the abovementioned cutoff values. Receiver operating curve (ROC) analysis and Youden’s index maxima were used to determine the optimal cutoff values that achieved optimal discrimination (Supplementary Table 4). Subcategories of IC as mobility (≥39.8 vs. <39.8), cognition (≥86.7 vs. <86.7), psychological (≥96.7 vs. <96.7), vitality (≥88.3 vs. <88.3), and sensory (≥83.3 vs. <83.3) were categorized into high and low for comparisons of baseline characteristics. Multivariable logistic regression was used to assess the associations between identified biomarkers and low IC. Cox proportional hazard regression was used to evaluate the association between IC, FA, and mortality. Schoenfeld residuals were used to test proportionality assumptions in Cox proportional hazard models. Kaplan–Meier survival plots and log-rank tests were applied to investigate the association between IC, FA, and mortality. In the second part of this study, multivariable logistic regressions were used to identify biomarkers associated with maintained or improved IC or rapidly deteriorated IC. The rapidly deteriorated IC group was defined as those with a decline in IC score >10% (equal to the 1 standard deviation (SD) lower than the group mean of decline in IC score) between wave 1 and 3 survey.

Biomarkers were dichotomized based on definitions used in previous literature: HOMA-IR (≥2 vs. <2) [45], high cholesterol (≥200 or taking lipid lower drugs vs. <200 mg/dL) [46], high triglycerides (≥150 or taking lipid lower drugs vs. <150 mg/dL) [46], high LDL-C (≥130 or taking lipid lower drugs vs. <130 mg/dL) [46], high fasting blood glucose (≥100 or taking oral anti-diabetic drugs vs. <100 mg/dL) [46], hyperhomocysteinemia (>15 vs. ≤15 μmol/L), and hsCRP (≥0.3 vs. <0.3 mg/dL) [47]. All other biomarkers, including the FAI, were tertilized [44]. Vitamin D insufficiency and deficiency were defined by 25-OH vitamin D <20 and <10 ng/mL, respectively [29].

A *p* value from two-sided tests <0.05 and 95% CIs not spanning the null hypothesis values were considered to be statistically significant. All analyses were performed using the SAS statistical package version 9.4 (SAS Institute, Inc., Cary, NC, USA).

## Supplementary Materials

Supplementary Figures

Supplementary Tables 1, 3 and 4

Supplementary Table 2
