# Supplementary material for: Intrinsic capacity differs from functional ability in predicting 10-year mortality and biological features in healthy aging: results from the I-Lan longitudinal aging study
Source: Aging (Albany NY). 2023 Feb 6;15(3):748–64. doi: 10.18632/aging.204508 (PMC9970311; doi:10.18632/aging.204508)
Supplement: Supplementary Figures [file aging-15-204508-s001.pdf]

SUPPLEMENTARY FIGURES

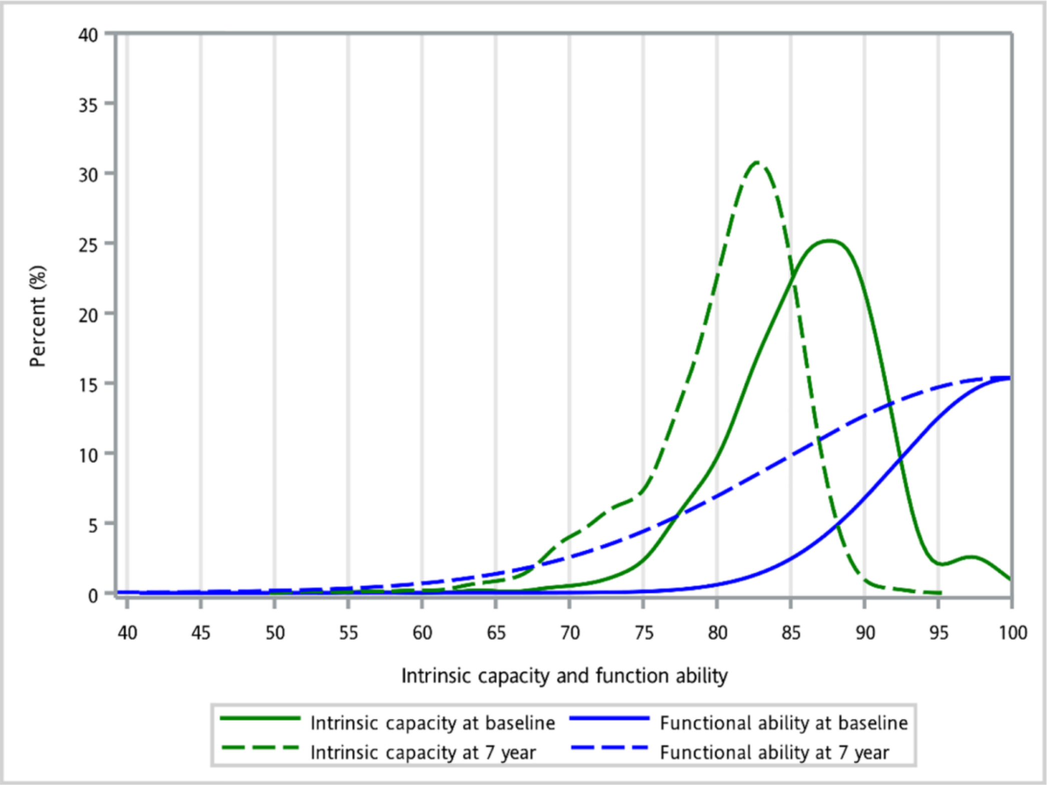

Supplementary Figure 1. Distribution of baseline and 7-year intrinsic capacity and functional ability.

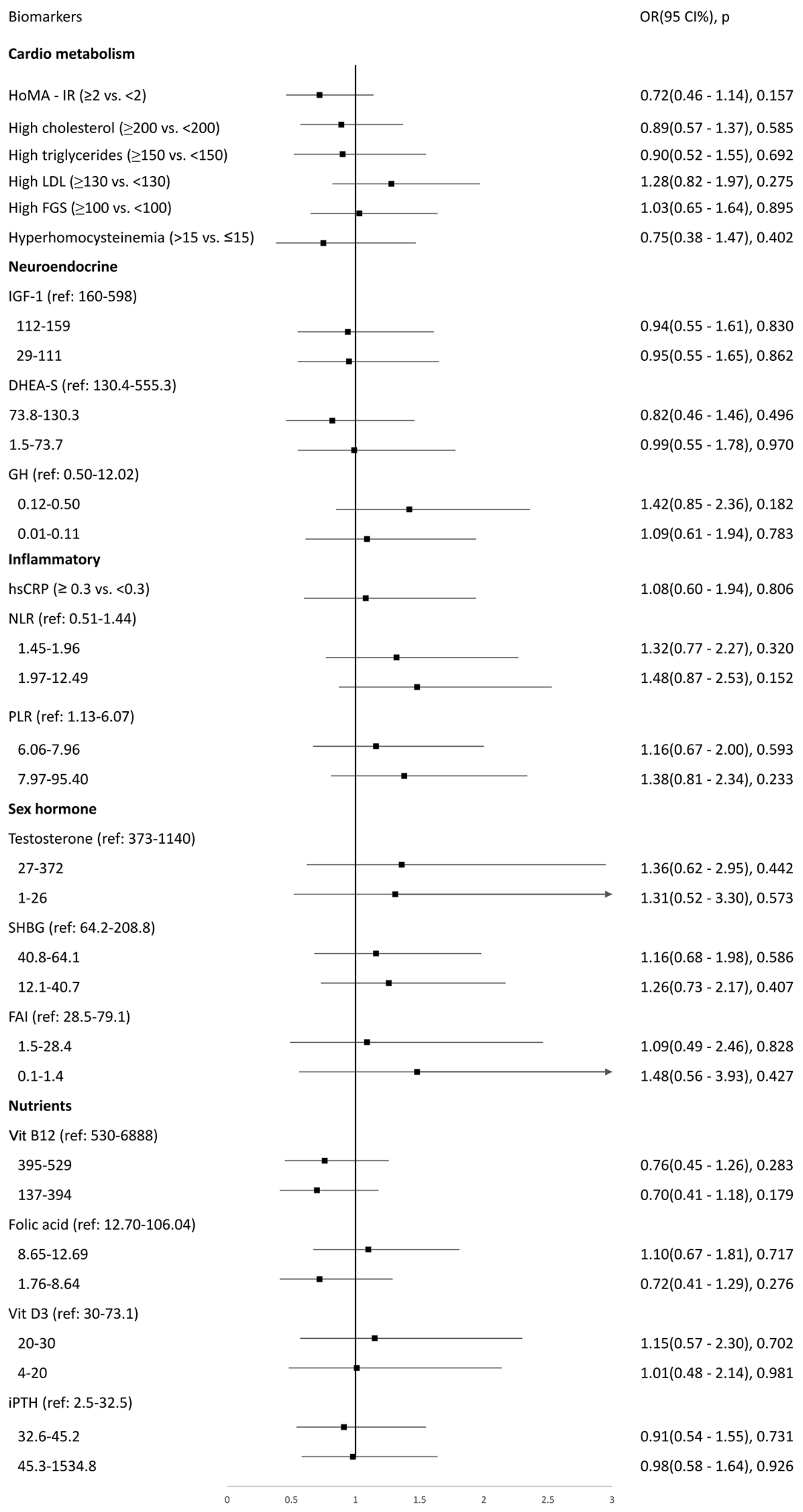

Supplementary Figure 2. Logistic regression to explore biomarkers associated with maintained and improved intrinsic capacity at a 7-year follow up period.
