# Supplementary material for: Intrinsic capacity differs from functional ability in predicting 10-year mortality and biological features in healthy aging: results from the I-Lan longitudinal aging study
Source: Aging (Albany NY). 2023 Feb 6;15(3):748–64. doi: 10.18632/aging.204508 (PMC9970311; doi:10.18632/aging.204508)
Supplement: Supplementary Tables 1, 3 and 4 [file aging-15-204508-s002.pdf]

SUPPLEMENTARY TABLES

Supplementary Table 1. Basic characteristics of participants by intrinsic capacity and functional ability.

Characteristics: mean ± SD or number (%)		Intrinsic capacity			Functional ability		
	Total (n = 1839)	High (n = 1190)	Low (n = 649)	p	High (n = 1769)	Low (n = 70)	p
Age (years)	63.9 ± 9.3	60.6 ± 7.7	70.0 ± 8.8	<0.001	63.5 ± 8.9	76.0 ± 9.9	<0.001
Sex (Men)	873 (47.5)	633 (53.2)	240 (37.0)	<0.001	835 (47.2)	38 (54.3)	0.244
Education (years)	6.2 ± 5.0	8.2 ± 4.5	2.5 ± 3.4	<0.001	6.4 ± 4.9	2.9 ± 4.1	<0.001
Current tobacco smoker[a]	336 (18.3)	220 (18.5)	116 (17.9)	0.745	320 (18.1)	16 (22.9)	0.311
Current alcohol consumer[a]	607 (33.0)	458 (38.5)	149 (23.0)	<0.001	596 (33.7)	11 (15.7)	0.001
Charlson comorbidity index	1.0 ± 1.3	0.7 ± 1.1	1.6 ± 1.4	<0.001	1.0 ± 1.2	2.3 ± 1.6	<0.001
Walking speed (m/s)	1.5 ± 0.5	1.7 ± 0.4	1.1 ± 0.3	<0.001	1.5 ± 0.5	1.0 ± 0.4	<0.001
MMSE	25.6 ± 4.0	27.6 ± 2.1	22.0 ± 4.2	<0.001	25.8 ± 3.8	21.1 ± 5.9	<0.001
CESD	2.4 ± 4.5	1.2 ± 2.2	4.5 ± 6.5	<0.001	2.2 ± 4.3	7.1 ± 7.4	<0.001
Mini-nutrition assessment	27.2 ± 1.8	27.7 ± 1.4	26.2 ± 2.1	<0.001	27.2 ± 1.7	25.7 ± 2.6	<0.001
Sensory score	0.0 ± 0.2	0.0 ± 0.1	0.0 ± 0.3	<0.001	0.0 ± 0.0	−0.5 ± 0.7	<0.001
SMAF	−0.2 ± 1.6	0.0 ± 0.1	−0.5 ± 2.7	<0.001	0.0 ± 0.0	−4.6 ± 7.0	<0.001
Intrinsic capacity	84.2 ± 6.2	87.8 ± 3.4	77.7 ± 4.8	<0.001	84.6 ± 5.7	73.5 ± 8.4	<0.001
Functional ability	99.8 ± 1.9	100.0 ± 0.1	99.4 ± 3.1	<0.001	100.0 ± 0.0	94.7 ± 8.1	<0.001

[a]Any use during 6 months preceding enrollment. Abbreviations: MMSE denotes: Mini-Mental State Examination; CESD denotes: Center for Epidemiologic Studies—Depression scale; SMAF denotes: Functional Autonomy Measurement System.

Supplementary Table 3. Details of measurement devices for serum biomarkers.

Biomarkers	Unit	Methods	Machine	Company	Country	Limit of detection	Intra- assay coefficients of variation	Inter-assay coefficients of variation
Fasting glucose	mg/dL	Hexokinase	ADVIA Chemistry XPT	SIEMENS	Germany	70	1.46	1.19
Insulin	mU/L	Chemiluminescence	ADVIA CentaurXPT	SIEMENS	Germany	3	4.79	4.17
Cholesterol	mg/dL	Enzymatic	ADVIA Chemistry XPT	SIEMENS	Germany	0	1.51	0.92
Triglycerides	mg/dL	Enzymatic GPO	ADVIA Chemistry XPT	SIEMENS	Germany	0	2	1.22
LDL	mg/dL	Direct Homogenous Surfactant	ADVIA Chemistry XPT	SIEMENS	Germany	0	2.54	1.68
Homocysteine	μmol/L	Chemiluminescence	ADVIA CentaurXPT	SIEMENS	Germany	6.5	5.52	5.24
IGF-1	ng/mL	Chemiluminescence Immunoassay	DOC Immulite 2000 Xpi	SIEMENS	Germany	0	5.11	5.73
DHEA-S	μg/dL	Electrochemiluminescense immunoassay (ECLIA)	Cobas e411	Roche	Germany	9.4	3.95	2.74
GH	ng/mL	Chemiluminescence	Access 2	Beckman Coulter	USA	0.1	3.444	2.429
hsCRP	mg/dL	Latex enhanced immunoturbidimetric	ADVIA Chemistry XPT	SIEMENS	Germany	0	2.898	1.251
White blood cell	$10^3/\mu L$	Microscopy	XN-9000	SYSMEX	Japan	3.5	1.21	1.19
Platelet	$10^3/\mu L$	Microscopy	XN-9000	SYSMEX	Japan	150	2.96	1.09
Neutrophil	%	Microscopy	XN-9000	SYSMEX	Japan	39	2.36	1.24
Lymphocyte	%	Microscopy	XN-9000	SYSMEX	Japan	19	3.36	1.76
Testosterone	ng/dL	Chemiluminescence	ADVIA CentaurXPT	SIEMENS	Germany	10	6	4.48
SHBG	nmol/L	Electrochemiluminescense immunoassay (ECLIA)	Cobas e411	Roche	Germany	0.35	3.99	3.6

Vitamin B12	pg/mL	Chemiluminescence	ADVIA CentaurXPT	SIEMENS	Germany	247	3.84	3.66
Folic acid	ng/mL	Chemiluminescence	ADVIA CentaurXPT	SIEMENS	Germany	5.39	8.06	7.95
25-OH vitamin D	ng/mL	Chemiluminescence	LIAISON	DiaSorin	Italy	30	3.77	4.01
iPTH	pg/mL	Chemiluminescence	ADVIA CentaurXPT	SIEMENS	Germany	18.5	5	2.86

Abbreviations: LDL denotes: low density lipoprotein cholesterol; IGF-1 denotes: insulin-like growth factor 1; DHEA-S denotes: dehydroepiandrosterone sulfate; GH denotes: growth hormone; hsCRP denotes high sensitive C reactive protein; SHBG denotes: sex hormone-binding globulin; iPTH denotes: intact parathyroid hormone.

Supplementary Table 4. ROC analysis to determine the cut-off values of intrinsic capacity and functional ability.

	Intrinsic capacity	Functional ability
c-statistics	0.710	0.546
Estimated coefficient	−0.114	−0.136
Standard Error	0.011	0.038
Odds Ratios (95% Confidence intervals)	0.89 (0.87,0.91)	0.87 (0.81,0.94)
Cutoff values	82.7	98.9
Sensitivity	0.634	0.122
Specificity	0.690	0.974
