# Supplementary material for: Intrinsic capacity differs from functional ability in predicting 10-year mortality and biological features in healthy aging: results from the I-Lan longitudinal aging study
Source: Aging (Albany NY). 2023 Feb 6;15(3):748–64. doi: 10.18632/aging.204508 (PMC9970311; doi:10.18632/aging.204508)
Supplement: Supplementary Table 2 [file aging-15-204508-s003.docx]

| **Supplementary Table 2. Basic characteristics of participants by subcategories of intrinsic capacity.** | | | | | | | | | | | | | | | |
| --- | --- | --- | --- | --- | --- | --- | --- | --- | --- | --- | --- | --- | --- | --- | --- |
| Characteristics: mean ± SD or number (%) | Mobility | | | Cognition | | | Psychological | | | Vitality | |  | Sensory | |  |
|  | High (n=1230) | Low (n=609) | p | High (n=996) | Low (n=843) | p | High (n=1172) | Low (n=667) | p | High (n=1394) | Low (n=445) | p | High (n=1834) | Low (n=5) | p |
| Age (years) | 61.2±8.1 | 69.4±9.1 | <0.001 | 60.0±7.6 | 68.5±9.0 | <0.001 | 63.3±9.1 | 65.1±9.5 | <0.001 | 63.2±8.9 | 66.3±10.0 | <0.001 | 63.9±9.2 | 84.1±7.7 | <0.001 |
| Sex(Men) | 640(52.0) | 233(38.3) | <0.001 | 517(51.9) | 356(42.2) | <0.001 | 598(51.0) | 275(41.2) | <0.001 | 705(50.6) | 168(37.8) | <0.001 | 870(47.4) | 3(60.0) | 0.673 |
| Education (years) | 7.7±4.8 | 3.2±3.8 | <0.001 | 9.0±4.1 | 3.0±3.7 | <0.001 | 6.9±5.1 | 5.1±4.6 | <0.001 | 6.6±5.0 | 4.9±4.6 | <0.001 | 6.2±5.0 | 1.6±2.3 | 0.037 |
| Current tobacco smoker^a^ | 994(80.8) | 509(83.6) | 0.148 | 815(81.8) | 688(81.6) | 0.906 | 973(83.0) | 530(79.5) | 0.057 | 1146(82.2) | 357(80.2) | 0.346 | 1499(81.7) | 4(80.0) | 0.999 |
| Current alcohol consumer^a^ | 765(62.2) | 467(76.7) | <0.001 | 616(61.8) | 616(73.1) | <0.001 | 777(66.3) | 455(68.2) | 0.400 | 897(64.3) | 335(75.3) | <0.001 | 1228(67.0) | 4(80.0) | 0.999 |
| Charlson comorbidity index | 0.8±1.1 | 1.5±1.4 | <0.001 | 0.7±1.1 | 1.4±1.3 | <0.001 | 0.9±1.2 | 1.2±1.4 | <0.001 | 0.9±1.2 | 1.5±1.4 | <0.001 | 1.0±1.3 | 3.4±1.7 | <0.001 |
| Walking speed (m/s) | 1.8±0.4 | 1.0±0.2 | <0.001 | 1.7±0.5 | 1.3±0.4 | <0.001 | 1.6±0.5 | 1.4±0.4 | <0.001 | 1.6±0.5 | 1.4±0.5 | <0.001 | 1.5±0.5 | 0.9±0.2 | 0.006 |
| MMSE | 26.9±3.0 | 23.1±4.6 | <0.001 | 28.5±1.1 | 22.2±3.6 | <0.001 | 26.1±3.8 | 24.8±4.3 | <0.001 | 26.0±3.7 | 24.4±4.7 | <0.001 | 25.7±4.0 | 14.2±7.0 | <0.001 |
| CESD | 1.7±3.2 | 3.8±6.2 | <0.001 | 1.8±3.7 | 3.1±5.3 | <0.001 | 0.3±0.5 | 6.1±5.9 | <0.001 | 1.8±3.4 | 4.3±6.6 | <0.001 | 2.4±4.5 | 14.4±8.5 | 0.034 |
| Mini-nutrition assessment | 27.4±1.6 | 26.7±2.0 | <0.001 | 27.5±1.5 | 26.8±2.0 | <0.001 | 27.5±1.6 | 26.6±2.1 | <0.001 | 28.0±1.0 | 24.7±1.6 | <0.001 | 27.2±1.8 | 23.9±3.5 | 0.105 |
| Sensory score | 0.0±0.1 | 0.0±0.2 | 0.001 | 0.0±0.1 | 0.0±0.2 | 0.001 | 0.0±0.1 | 0.0±0.2 | <0.001 | 0.0±0.1 | 0.0±0.2 | 0.047 | 0.0±0.1 | -2.2±0.4 | <0.001 |
| SMAF | 0.0±0.1 | -0.5±2.8 | <0.001 | 0.0±0.7 | -0.3±2.3 | <0.001 | 0.0±0.3 | -0.4±2.7 | <0.001 | -0.1±0.6 | -0.5±3.1 | 0.002 | -0.1±1.2 | -15.7±14.3 | 0.071 |
| Intrinsic capacity | 87.1±4.2 | 78.4±5.4 | <0.001 | 87.7±4.0 | 80.1±5.9 | <0.001 | 86.0±5.2 | 81.0±6.6 | <0.001 | 85.5±5.3 | 80.1±7.1 | <0.001 | 84.3±6.1 | 59.2±8.4 | <0.001 |
| Functional ability | 100.0±0.2 | 99.4±3.2 | <0.001 | 99.9±0.9 | 99.6±2.6 | <0.001 | 100.0±0.3 | 99.5±3.1 | <0.001 | 99.9±0.7 | 99.4±3.5 | 0.002 | 99.8±1.4 | 82.0±16.4 | 0.071 |
| ^a^Any use during 6 months preceding enrollment. MMSE denotes Mini-Mental State Examination; CESD denotes Center for Epidemiologic Studies—Depression scale; SMAF denotes Functional Autonomy Measurement System.. | | | | | | | | | | | | | | | |
